# Supplementary material for: Is Satisfaction with Online Learning Related to Depression, Anxiety, and Insomnia Symptoms? A Cross-Sectional Study on Medical Undergraduates in Romania
Source: Eur J Investig Health Psychol Educ. 2023 Mar 10;13(3):580–94. doi: 10.3390/ejihpe13030045 (PMC10046999; doi:10.3390/ejihpe13030045)
Supplement: Supplementary file 1 [file ejihpe-13-00045-s001.zip › ejihpe-2216675-supplementary.pdf]

Table S1 (Supplementary Materials- All the scales used in the study)

- Patient Health Questionnaire (PHQ-9).

**Over the <u>last 2 weeks</u>, how often have you been bothered by any of the following problems?** *(Use "✔" to indicate your answer)*	**Not at all**	**Several days**	**More than half the days**	**Nearly every day**
**1.** Little interest or pleasure in doing things	0	1	2	3
**2.** Feeling down, depressed, or hopeless	0	1	2	3
**3.** Trouble falling or staying asleep, or sleeping too much	0	1	2	3
**4.** Feeling tired or having little energy	0	1	2	3
**5.** Poor appetite or overeating	0	1	2	3
**6.** Feeling bad about yourself — or that you are a failure or have let yourself or your family down	0	1	2	3
**7.** Trouble concentrating on things, such as reading the newspaper or watching television	0	1	2	3
**8.** Moving or speaking so slowly that other people could have noticed? Or the opposite — being so fidgety or restless that you have been moving around a lot more than usual	0	1	2	3
**9.** Thoughts that you would be better off dead or of hurting yourself in some way	0	1	2	3

- Generalized Anxiety Disorder-7 (GAD-7)

Over the last 2 weeks, how often have you been bothered by the following problems?	Not at all sure	Several days	Over half the days	Nearly every day
1. Feeling nervous, anxious, or on edge	0	1	2	3
2. Not being able to stop or control worrying	0	1	2	3
3. Worrying too much about different things	0	1	2	3

4. Trouble relaxing	0	1	2	3
5. Being so restless that it's hard to sit still	0	1	2	3
6. Becoming easily annoyed or irritable	0	1	2	3
7. Feeling afraid as if something awful might happen	0	1	2	3
Add the score for each column	+	+	+	
Total Score *(add your column scores)* =				

- Insomnia Severity Index (ISI)

1. Please rate the current (i.e., last week) **SEVERITY** of your insomnia problem(s).

<30 min **30-45** **45-90** **90-120** **>120**

	None	Mild	Moderate	Severe	Very
Difficulty falling asleep:	0	1	2	3	4
Difficulty staying asleep:	0	1	2	3	4
Problem waking up too early:	0	1	2	3	4

2. How **SATISFIED**/dissatisfied are you with your current sleep pattern?

Very Satisfied				Very Dissatisfied
0	1	2	3	4

3. To what extent do you consider your sleep problem to **INTERFERE** with your daily functioning (e.g. daytime fatigue, ability to function at work/daily chores, concentration, memory, mood, etc.).

Not at all Interfering A Little Somewhat Interfering Much Very Much

0 1 2 3 4

4. How **NOTICEABLE** to others do you think your sleeping problem is in terms of impairing the quality of your life?

Not at all Noticeable		Barely	Somewhat	Much	Very Much Noticeable
0	1	2		3	4

5. How **WORRIED/**distressed are you about your current sleep problem?

Not at all	A Little	Somewhat	Much	Very Much
0	1	2	3	4

- The perception of online learning satisfaction was assessed with a 20-item questionnaire which was used based on sections I to IV of the Dundee Ready Edu-cation Environment Measure (DREEM)

Online Learning & Medical Education

4. Prior to the Covid-19 pandemic, which online learning platforms/resources did you engage with? (Please select all which apply)

- ☐ Video tutorials e.g. Youtube/Osmosis
- ☐ Live tutorials via Zoom/similar platforms by **Medical School**
- ☐ Live tutorials via Zoom/similar platforms by **other sources**
- ☐ Online question banks
- ☐ Online/Digital Flashcards e.g. Brainscape, Anki
- ☐ Pre-recorded tutorials via Medical School specific online learning platform
- ☐ None
- ☐ Other - please specify

5. Which method of online learning do you find the most effective? Please rank the following methods from 1-5 (1=most effective, 5=least effective)

- Video tutorials e.g. Youtube/Osmosis
- Live tutorials via Zoom/similar platforms
- Online question banks
- Online/Digital Flashcards e.g. Brainscape, Anki
- Other - please specify:

6. Prior to the Covid-19 pandemic, how many hours per week did you spend on average on online learning?

7. During the current Covid-19 pandemic, how has your medical school adapted teaching for your year? (Please select all which apply)

- ☐ Introduced a new online learning platform with new resources
- ☐ Introduced new resources to an existing online learning platform
- ☐ Delivered live tutorials via Zoom/similar platforms
- ☐ Delivered pre-recorded tutorials
- ☐ Other - please specify:

8. Are these online teaching sessions interactive?

- ○ Yes
- ○ No
- ○ Majority are
- ○ Majority are not

8b. What makes your teaching sessions interactive? (Please select all which apply)

- ☐ Opportunity to interact via chat box
- ☐ Opportunity to interact via speech
- ☐ Live quiz

☐ Other - please specify:

9. Does the online learning follow a pre-set curriculum, or is it based on student requests?

✓
Pre-set curriculum
Student requests
Combination of both

10. During the current Covid-19 pandemic, how many hours per week do you spend on average on online learning?

Student Perceptions of Online Learning

11. Please rank the following statements on your experience of online learning from 1-5 (1=Strongly disagree, 5 = Strongly agree)

0 1 2 3 4 5

The teaching is often stimulating

I find it easy to engage in the lesson

I feel able to ask the questions I want

I enjoy the online teaching

I would like the online teaching to be more interactive

I feel that online teaching is as effective as face-face teaching

I prefer online teaching to face-face teaching

The teachers are well prepared for the teaching sessions

I feel I am being well prepared for my profession

My internet connection can be problematic

12. What aspects of online learning do you enjoy? (Please select all which apply)

- ☐ No travel
- ☐ Ability to ask questions
- ☐ Cost savings
- ☐ More comfortable
- ☐ Interactive
- ☐ Ability to learn at own pace

☐ Flexibility

☐ Other - please specify:

13. What do you feel are the barriers to online learning? (Please select all which apply)

☐ Internet connection

☐ Lack of space

☐ Timing of tutorials

☐ Lack of devices

☐ Family distractions

☐ Anxiety

☐ Other - please specify:

Role of Online Learning in Clinical Teaching

14a. Do you feel online learning has successfully replaced the clinical teaching you receive from direct patient contact?

✓
Yes
No
Yes, to some extent
N/A

14b. Do you feel able to learn practical clinical skills through online learning?

✓
Yes
No
Yes, to some extent
N/A

15. Have your examinations been affected by Covid-19?

○ Yes

○ No

○ N/A

- provided information about the average num-ber of hours spent online for studying, doing job or internship-related work, watch-ing TV, using social media, watching video classes, reading, doing research or schoolwork, time spend playing video games on games console, computer, television, tablet, smartphone

On a typical day, how much time do you spend studying, watching video classes, reading, doing research or school work on a computer, television, tablet, smartphone?

On a typical day, how much time do you spend studying in total?

On a typical day, how much time do you spend doing job or internship-related work on a computer, television, tablet, smartphone?

On a typical day, how much time do you spend watching TV shows, movies, soap operas, news, sports,programs, Twitch, podcasts or other videos on a computer, television, tablet, smartphone

On a typical day, how much time do you spend playing video games on a games console, computer, television, tablet, smartphone

On a typical day, how much time do you spend using social media like Facebook, Instagram, Twitter, Snapchat, or chat applications like WhatsApp, Telegram, Messenger on a computer, television, tablet, smartphone

Respnonses: 0-30 minutes, 1-3 hours, >3 hours
